# Interactions between a fungal entomopathogen and malaria parasites within a mosquito vector

**DOI:** 10.1186/s12936-014-0526-x

**Published:** 2015-01-28

**Authors:** Rebecca L Heinig, Matthew B Thomas

**Affiliations:** Merkle Laboratory, The Pennsylvania State University, University Park, PA 16803 USA

**Keywords:** *Plasmodium yoelii*, *Plasmodium falciparum*, *Anopheles stephensi*, *Beauveria bassiana*, Biological control

## Abstract

**Background:**

Mosquitoes are becoming increasingly resistant to the chemical insecticides currently available for malaria vector control, spurring interest in alternative management tools. One promising technology is the use of fungal entomopathogens. Fungi have been shown to impact the potential for mosquitoes to transmit malaria by reducing mosquito longevity and altering behaviour associated with flight and host location. Additionally, fungi could impact the development of malaria parasites within the mosquito via competition for resources or effects on the mosquito immune system. This study evaluated whether co-infection or superinfection with the fungal entomopathogen *Beauveria bassiana* affected malaria infection progress in *Anopheles stephensi* mosquitoes.

**Methods:**

The study used two parasite species to examine possible effects of fungal infection at different parasite development stages. First, the rodent malaria model *Plasmodium yoelii* was used to explore interactions at the oocyst stage. *Plasmodium yoelii* produces high oocyst densities in infected mosquitoes and thus was expected to maximize host immunological and resource demands. Second, fungal interactions with mature sporozoites were evaluated by infecting mosquitoes with the human malaria species *Plasmodium falciparum*, which is highly efficient at invading mosquito salivary glands.

**Results:**

With *P. yoelii*, there was no evidence that fungal co-infection (on the same day as the blood meal) or superinfection (during a subsequent gonotrophic cycle after parasite infection) affected the proportion of mosquitoes with oocysts, the number of oocysts per infected mosquito or the number of sporozoites per oocyst. Similarly, for *P. falciparum*, there was no evidence that fungal infection affected sporozoite prevalence. Furthermore, there was no impact of infection with either malaria species on fungal virulence as measured by mosquito survival time.

**Conclusions:**

These results suggest that the impact of fungus on malaria control potential is limited to the well-established effects on mosquito survival and transmission behaviour. Direct or indirect interactions between fungus and malaria parasites within mosquitoes appear to have little additional influence.

**Electronic supplementary material:**

The online version of this article (doi:10.1186/s12936-014-0526-x) contains supplementary material, which is available to authorized users.

## Background

Malaria vector control programmes are currently being threatened by increasing insecticide resistance in adult mosquito populations [1-4]. This has spurred interest in the development of alternative management tools, including the use of entomopathogenic fungi [5-8]. Fungal spores infect mosquitoes on contact and can be integrated into a number of delivery systems, including wall treatments [7,9-11], eave nets and curtains [8], baited traps [12], and point source targets [8,13,14]. Conidial storage and persistence characteristics are competitive with those of commonly-used chemical insecticides [10]. Importantly, fungi are effective against existing insecticide-resistant mosquito populations [6,7,15] and are expected to impose reduced selection for new resistance traits relative to conventional chemicals [16-19].

Chemical insecticides generally kill or knock down susceptible mosquitoes within hours of exposure [20]. In contrast, fungal biopesticides are relatively slow-acting, taking up to a week or more to kill exposed mosquitoes [5,13]. This slower action can still be sufficient to block malaria transmission, since mosquitoes do not become infective until the end of the malarial extrinsic incubation period (EIP) of approximately two weeks. Fungal infection also causes a number of sublethal effects (e.g., decreased host location ability [21] and feeding propensity [7,11,22,23]) that further reduce the probability of disease transmission. One early study found that the combination of high mortality and low sporozoite rates associated with *Beauveria bassiana* fungal infection significantly reduced the number of *Anopheles stephensi* mosquitoes that both survived to the end of the EIP and were potentially able to transmit *Plasmodium chabaudi* malaria [5]. The authors also noted that mosquitoes co-infected with fungus and malaria exhibited an upward trend in daily mortality rate toward the end of the EIP that was not evident in mosquitoes infected with either malaria or fungus alone [5]. However, there has been negligible follow-up work to validate this preliminary observation. If either co-infection (i.e., mosquitoes infected with fungus and malaria parasites at more or less the same time during the same gonotrophic cycle) or superinfection (i.e., mosquitoes encountering the fungus during a later gonotrophic cycle when the malaria parasite is already established) alter the development of either the fungus or malaria parasite, there could be important implications for malaria control. For example, if fungal infection directly or indirectly inhibited sporozoite invasion of the salivary glands, control programmes could potentially use fungal strains that were less virulent to mosquitoes, which would in turn reduce selection for resistance in vector populations [16-19].

The effects of co- and superinfection are highly variable in other mosquito-pathogen systems. The fungal entomopathogens *Metarhizium anisopliae* and *B. bassiana* can inhibit dengue virus replication and dissemination in co-infected *Aedes aegypti* mosquitoes [24,25]. Infection with certain species of *Wolbachia* bacteria also can inhibit establishment of other bacterial [26], nematode [26] and viral [27-32] superinfections. The impacts of *Wolbachia* infection on malaria parasites have been mixed, with reductions in oocyst densities occurring under some conditions [33-35] and enhancement observed under others [36,37]. The mechanisms underlying these phenotypes remain unresolved but appear to be mediated by resource competition [38] and/or upregulation of immune factors [24,27,28,34,35,39,40].

Similar mechanisms could affect interactions between fungal pathogens and malaria parasites. The mosquito responds to the early ookinete stages of malaria infection by upregulating immune responses including melanization [41] and the Toll pathway or, in the case of human malaria species, the IMD pathway [42-44]. There is evidence that malaria parasites utilize host resources as sporozoites replicate within the oocysts [45], and resource depletion might increase host susceptibility to secondary infection. During the final stage of infection, sporozoites are actively degraded in the haemocoel [46], potentially either reducing (via depletion) or enhancing (via upregulation) the availability of haemocytes to combat additional infectious agents. Fungal infection itself triggers a number of similar immune responses in insect hosts. Early fungal invasion of the haemocoel is countered by cellular immune responses [47], which can result in granulocyte depletion as the infection progresses [48]. Later in the infection, immune factors involved in the humoral melanization response [47,49] and the Toll and JAK-STAT pathways [24] are involved in countering fungal proliferation. Thus, depending on the timing of the malaria and fungal infections, there might be extensive overlap in immune and resource demands on the mosquito host.

This study explored whether co- or superinfection with a candidate strain of the fungal entomopathogen *B. bassiana* affected a number of malaria infection parameters. Two different malaria species were used to evaluate potential interactions at different stages of the malaria life cycle. To examine impacts at the oocyst stage, *A. stephensi* mosquitoes were infected with *Plasmodium yoelii*, a rodent malaria species which produces high oocyst densities in infected mosquitoes [50]. High oocyst intensities have been found to increase vector mortality rates [51-53] (but see [54]), so it was expected that any mortality costs associated with malaria-fungus co-infection would be maximized in high-intensity infections. The mosquitoes were exposed to fungus either immediately following the blood meal (to simulate co-infection) or three days later to simulate superinfection during the next gonotrophic cycle following oocyst establishment. There were no significant effects of co- or superinfection on oocyst prevalence, oocyst intensity, sporozoite replication (represented by the number of sporozoites per oocyst), or mosquito mortality rate among the various treatment groups.

To determine whether there were late-stage interactions between fungal superinfection and malaria sporozoites, mosquitoes were infected with *Plasmodium falciparum*, a human malaria species which produces lower oocyst densities but is much more efficient at invading mosquito salivary glands than *P. yoelii* [55,56]. Late fungal infection was simulated by exposing mosquitoes to fungus either eight or 11 days after the infectious blood meal. Regardless of exposure day, fungal treatment had no effect on sporozoite prevalence in the salivary glands, nor did the presence of malaria appear to affect subsequent fungal virulence. Overall, the results did not indicate a significant interaction between mosquito, fungus and parasite, and suggest that interaction with malaria infection is unlikely to have either positive or negative consequences for this potential of this fungal biopesticide to reduce transmission.

## Methods

### Mosquito rearing

*Anopheles stephensi* mosquitoes were raised under standard insectary conditions at 27°C and 75% relative humidity with a 12-hour light/12-hour dark cycle. Mosquito eggs were hatched in plastic tubs containing 1.5 l of distilled water. Four days later, larvae were placed into new tubs containing 400 individuals per tub and provided with 10 mg of powdered Tetrafin fish flakes (TetraFin, Melle, Germany) daily. Pupae were placed into cages for emergence, and adults were given a 10% glucose solution supplemented with 0.05% para-aminobenzoic acid (PABA) to enhance *P. yoelii* oocyst infection rate [57].

### Conidial production and formulation

Oil suspensions of *B. bassiana* (isolate I93-825) conidia were prepared according to established protocols [5,7]. Conidia harvested from potato dextrose agar (Oxoid, UK) were suspended in sterile 0.05% Tween 80 (Sigma) at a concentration of 10^6^ conidia/ml. Liquid cultures containing 1 ml of suspension and 75 ml of sterile liquid culture medium (4% d-glucose, 2% yeast extract (Oxoid, UK) in tap water) were incubated on a shaker at 24°C and 160 rpm for three days then diluted with 75 ml distilled water. The mixture was used to inoculate sterile solid medium (1 kg barley flakes (Bobs Red Mill, Milwaukie, OR, USA) and 600 ml tap water), sealed in mushroom spawn bags (Unicorn, Garland, TX, USA) and incubated at 24°C for ten days. The bag contents were then dried in paper bags to a moisture level of <20%. Conidia were harvested using a Mycoharvester (Acis Manufacturing, Devon, UK), dried over silica gel to a moisture level of 5% and sealed in foil sachets for storage at 5°C. Prior to the experiment, conidial viability was assessed by suspending conidia in Isopar M oil and plating the suspension on Sabouraud dextrose agar (Oxoid, UK). Three replicate plates were incubated at 25°C for 20 hours, and 300 spores per plate were visually assessed under a compound microscope to ensure that more than 85% of conidia had successfully germinated. A new suspension was prepared by adding dry conidia to an oil mixture (80% Isopar M:20% Ondina) at a concentration of 10^7^ conidia/ml, which was verified using a hemocytometer. The suspension was then applied to clay tiles as described below.

### Substrate preparation

The conidial suspensions were applied to clay tiles in a manner designed to simulate spray treatments on clay/mud walls of traditional African huts [7,10,20]. Tiles were created by pouring a slurry of white earthenware clay (Clay King, Spartanburg, SC, USA) and distilled water into 150 mm petri plates. All tiles were air-dried for at least one week until they had hardened completely. The dry tiles were then affixed to the back wall of a fume hood, and a handheld airbrush sprayer was used to uniformly apply 20 ml of conidial suspension to a 0.5 sq m area for a final application rate of 8 × 10^8^ conidia/sq m. At higher application rates, this fungal isolate kills mosquitoes within three to five days [7], so this relatively low application rate was selected to allow mosquitoes to live long enough post-infection so that potential interactions with malaria could be observed. Control tiles were sprayed with a blank oil formulation, and all tiles were air-dried overnight.

### *Plasmodium yoelii* assays

The rodent malaria model *P. yoelii* was used to assess the impact of fungal co- and superinfection on malaria oocyst prevalence and intensity. Three- to five-day-old female mosquitoes were starved overnight then allowed to feed on female six to eight week-old C57 mice (Charles River, Malvern, PA, USA) for up to 30 min. Mosquitoes from the malaria infection treatments were fed on anesthetized mice which had been injected with 10^5^*P. yoelii* parasites (*yoelii* strain, clone 17XNL, WHO Registry of Standard Malaria Parasites, University of Edinburgh, UK) four days prior, while mosquitoes from the control treatments were fed on uninfected mice. All blood feeds took place at 26°C to maximize feeding, and mosquitoes that were not fully engorged were removed from the experiment. The mosquitoes were then moved to a 24°C incubator to maximize *P. yoelii* growth and survival [58].

Following a one-hour acclimation period, the mosquitoes were randomly allocated to a number of treatment groups. Half the mosquitoes were exposed to fungus-treated or control tiles on the same day as the blood meal (day 0 exposures). Groups of approximately 50 individuals were aspirated into standard WHO cones on tiles and left for a 30-min exposure period. Afterward, the mosquitoes were aspirated into nylon-covered cups and placed in a 24°C incubator. Mortality was monitored daily. The other half of the mosquitoes were aspirated directly into nylon-covered cups and placed into the 24°C incubator. In this group, delayed fungal exposure was simulated by exposing the mosquitoes to the tiles three days after the blood feed, coinciding with when they would have been expected to seek their next blood meal (day 3 exposure). Thus, there were four treatment groups for each of the two exposure time points: dual infection (fungus and malaria), fungal infection alone, malaria infection alone and no infection. There were five replicate cups of approximately 50 mosquitoes per cup (~250 mosquitoes total) for each treatment and time point. All cups were held at 24°C and provided with a cotton ball saturated with glucose-PABA solution for nutrition. Mosquito mortality was monitored daily until all mosquitoes in the fungal treatments were dead (28 days in the day 0 exposures, 25 days in the day 3 exposures).

Seven days after the blood feed, 20 mosquitoes from each treatment group were dissected and examined for oocysts. Oocyst prevalence (the proportion of infected mosquitoes in each treatment) and oocyst intensity (the number of oocysts in each infected individual) were recorded, and each midgut was placed in 10 μl of 70% EtOH and stored at −80°C.

Quantitative PCR was used to estimate the number of sporozoites per oocyst, a measure of parasite replication rate. The Microelute Tissue DNA Kit (Omega Bio-tek) was used to extract and purify the DNA according to the manufacturer’s instructions with one exception: during the tissue lysis step, a stainless steel ball was added to each tube. The samples were then homogenized for 30 sec at 30 Hz on a TissueLyser (Qiagen) prior to being incubated at 55°C and processed according to the kit protocol. The purified DNA was eluted in 20 μL of buffer and stored at −20°C.

Real-time quantitative PCRs were performed using the *Plasmodium* primers and probe described by Bell *et al.* [59]. Reaction mixtures were prepared by adding 2 μL of purified DNA template to a reaction mix of 1.5 μL each of 5 μM dilutions of forward and reverse primers, 1 μL of 5 μM probe, 12.5 μL of PerfeCTa® qPCR FastMix® (UNG, Low ROX™ by Quanta BioSciences) and 6.5 μL of RNAse-free water. The reactions were run on a Prism 7500 Sequence Detection System (TaqMan) with an initial 20-sec activation step at 95°C followed by 40 cycles of denaturation at 95°C for 3 sec and annealing/extension at 60°C for 30 sec. Serial dilutions of *P. yoelii* DNA standard spanning five orders of magnitude (7.22 × 10^4^ to 7.22 sporozoites) were used to generate a standard curve for absolute quantification of the samples. Three replicates of each standard were included in each reaction run.

### *Plasmodium falciparum* assays

The human malaria parasite *P. falciparum* was used to assess interactions between fungal infection and sporozoites. *In vitro* gametocyte production followed established procedures [60]. NF54 strain *P. falciparum* cultures were maintained *in vitro* in O+ erythrocytes in a culture medium of RPMI 1640 (25 mM HEPES, 2 mM L-glutamine), 50 μM hypoxanthine and 10% A+ serum in an atmosphere of 5% CO_2_, 5% O_2_ and 90% N_2_. At 5% haematocrit and 0.8-1% parasitaemia (mixed stages), gametocyte cultures were initiated. Media was changed daily, and the cultures were maintained for up to 17 days. On the day of the feed, the gametocyte cultures were spun down, and the pelleted infected erythrocytes were diluted to 2% gametocytaemia and 40% haematocrit with fresh A+ human serum and O+ erythrocytes.

Three- to five-day-old female mosquitoes were aspirated into replicate cups and allowed to feed on warmed membrane feeders containing either the infectious blood mixture or uninfected blood for up to 30 min. Unfed mosquitoes were removed from each cup as described above. Seven days after the blood meal, seven mosquitoes from each malaria-infected cup were dissected as described above to ensure that there were no significant differences in initial oocyst prevalence (mean ± SE 0.44 ± 0.05, Fisher’s exact test p = 0.116) or intensity (1.52 ± 0.12; Kruskal-Wallis χ^2^ = 1.78, df = 3, p = 0.620) among the malaria treatment groups.

As described in the *P. yoelii* experiment, the mosquitoes were exposed to either fungus-treated or control tiles at two time points following the blood feed, generating four treatment groups (dual infection, fungal infection alone, malaria infection alone and no infection) per time point. However, this experiment simulated late fungal exposure by exposing the mosquitoes to the tiles either eight or 11 days after the blood feed. The day 11 treatments each included three replicate cups of approximately 30 mosquitoes per cup, and the day 8 treatments each included three replicate cups of approximately 50 mosquitoes per cup to ensure that an adequate number survived to the sporozoite stage. All cups were held at 27°C and were supplied with a cotton ball soaked with glucose-PABA solution, and daily mortality was monitored for 14 days following the blood meal. Over the next two days (days 15–16 after the blood meal), the remaining mosquitoes in the malaria infection treatments (malaria alone and dual infection) were dissected, and their salivary glands were inspected under a microscope for the presence of sporozoites. In the malaria control treatments (fungus alone and no infection), daily mortality was monitored until all the fungus-exposed mosquitoes had died (22 days after fungal exposure in the day 8 exposures, 20 days in the day 11 exposures).

### Statistical analysis

Median survival times for each treatment group were calculated using the Kaplan-Meier procedure. The effects of fungal exposure and malaria infection on mosquito survival were analysed using a full factorial Cox proportional hazards model with backward stepwise elimination of non-significant interactions (p > 0.05). The impacts of fungal exposure and malaria infection on the various malaria infection parameters were evaluated using generalized linear models (GLM) with the model error distributions and link functions adjusted to fit the data. For the *P. yoelii* experiments, oocyst prevalence was analysed using a binomial GLM with a logit link function. Oocyst intensity and the mean number of sporozoites per oocyst were heavily right-skewed, so treatments were compared using a negative binomial GLM with a log link function. In the *P. falciparum* experiments, the data for sporozoite prevalence (the proportion of dissected mosquitoes with sporozoites in their salivary glands) were overdispersed, so treatment effects were analysed using a quasibinomial GLM with a logit link function. In each case, the analyses began with a full factorial model including malaria infection and fungal exposure as variables. In the *P. falciparum* experiments, replicate cup was also included in the initial model but was not significant. Non-significant variables (p > 0.05) were then removed from each model using backward stepwise elimination. All analyses were performed in R [61].

## Results

### *Plasmodium yoelii* experiment

**Figure 1:**
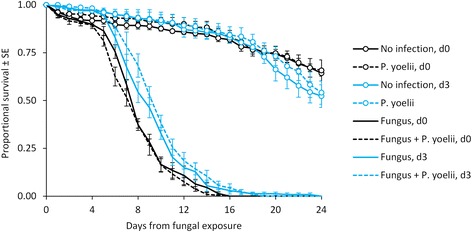
**Cumulative proportional survival of adult mosquitoes in the** ***Plasmodium yoelii*** **experiment.** *Anopheles stephensi* mosquitoes were fed either on *P. yoelii-*infected or control mice (indicated by line type), then exposed to tiles sprayed with *B. bassiana* conidial suspensions or blank oil (indicated by marker) either the same day as the blood meal (d0) or three days later (d3, indicated by line colour). Each point represents the mean of five replicates (± standard error).

Mosquito mortality rate varied with treatment group (Figure 1) but was not significantly affected by *P. yoelii* malaria infection status (hazard ratio (HR) = 0.95, z = −0.77, p = 0.44). Fungal exposure significantly increased mortality rate (HR = 17.06, z = 21.11, p < 0.001); mosquitoes exposed to fungus had median survival times of eight to ten days relative to >25 days in the controls. There was also a significant interaction between fungal exposure and exposure day (HR = 0.64, z = −3.42, p = 0.001), such that, even after accounting for the delay in fungal infection, the mosquitoes exposed to fungus on day 3 had median survival times about one day longer than those exposed on the same day as the blood meal.

**Figure 2:**
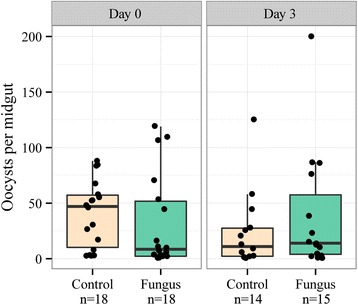
***Plasmodium yoelii*** **oocyst intensity (number of oocysts per midgut).** Boxes represent the first and third quartiles, and whiskers encompass the values within 1.5 interquartile ranges of the lower and upper quartiles.

**Figure 3:**
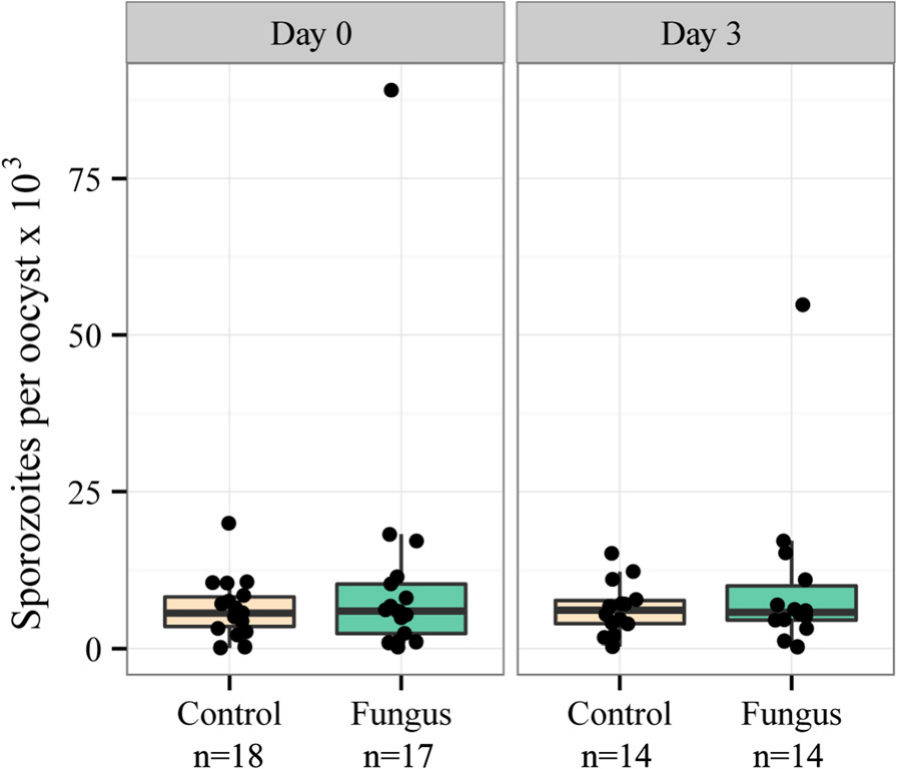
**Number of** ***Plasmodium yoelii*** **sporozoites per oocyst (10** ^**3**^ **) by treatment.** Boxes represent the first and third quartiles, and whiskers encompass the values within 1.5 interquartile ranges of the lower and upper quartiles.

None of the *P. yoelii* malaria infection parameters was significantly affected by fungal exposure or exposure timing (Table 1, Figures 2 and 3). Infection prevalence was slightly higher in the day 0 exposure groups (90% for both fungus and control mosquitoes) than in the day 3 groups (75% for fungus and 70% for controls), but the difference was marginally insignificant (z = −1.94, p = 0.053). Although the number of sporozoites per oocyst was lower in mosquitoes exposed to fungus (z = 2.15, p = 0.032), this result was driven by a single outlier. When this data point was not included in the analysis, neither fungal exposure (χ^2^ = 1.22, p = 0.269) nor exposure timing (χ^2^ = 1.06, p = 0.303) significantly affected sporozoite density per oocyst.

**Table 1:** **Summary of** ***P. yoelii*** **malaria infection parameters**

**Exposure day**	**Fungal treatment**	**Oocyst prevalence**	**Oocyst intensity**			**Sporozoites per oocyst (×10** ^**3**^ **)**		
			**N**	**Mean ± SE**	**Median**	**N**	**Mean ± SE**	**Median**
Day 0	Fungus	0.9	18	31.72 ± 9.93	8.5	17	11.44 ± 5.01	5.93
	Control	0.9	18	40.67 ± 7.07	47	18	6.38 ± 1.10	5.60
Day 3	Fungus	0.75	15	38.33 ± 14.05	14	14	10.10 ± 3.67	5.71
	Control	0.7	14	24.29 ± 9.07	11	14	6.35 ± 1.12	6.06

Oocyst prevalence represents the proportion of *An. stephensi* mosquitoes with ≥1 oocyst in a sample of n = 20 mosquitoes per treatment. Of the infected mosquitoes (N), we report the mean (± standard error) and median estimates for oocyst intensity and number of sporozoites per oocyst. The sample number decreases slightly in the sporozoite per oocyst because the PCR failed for some of the midgut samples.

### *Plasmodium falciparum* experiment

**Figure 4:**
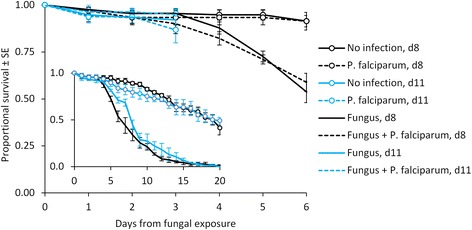
**Cumulative proportional survival of adult mosquitoes in the** ***Plasmodium falciparum*** **experiment.** *Anopheles stephensi* mosquitoes were fed on control mice, then exposed to tiles sprayed with *B. bassiana* conidial suspensions (‘fungus’) or blank oil (indicated by marker) either eight (d8) or 11 days later (d11, indicated by line colour). Each point represents the mean of three replicates (± standard error). (*Inset*) Proportional survival (y-axis) of malaria control treatments (*B. bassiana* fungus only and no infection groups) through day 20 following fungal exposure (x-axis).

There was no evidence that mosquito survival to the infectious stage (14 days after the blood feed) was affected by *P. falciparum* infection (HR = 1.02, z = 0.14, p = 0.888). There was a significant interaction between fungal exposure and exposure day (HR = 5.57, z = 2.90, p = 0.004), but this was likely due to the timing of the dissections. Mosquito survival is generally quite high in the first few days of fungal infection because the fungus requires time to develop and invade the haemocoel [47]. Once the infection is established, however, mosquito survival plummets rapidly. Mosquitoes in the day 11 malaria treatments were dissected just three days after fungal exposure when very few mosquitoes had died (≤14% in all treatments, Figure 4). In contrast, mosquitoes in the day 8 treatments were dissected six days after fungal exposure, at which point mosquito mortality was much higher in the fungal treatment groups (>40%) than the controls (<10%). When only the first three days following exposure were evaluated in all the treatments, neither exposure day (HR = 0.98, z = −0.06, p = 0.95) nor fungal exposure (HR = 1.14, z = 0.34, p = 0.71 significantly influenced mortality. There was also no evidence that exposure day significantly affected mortality rate in the malaria control treatments (HR = 1.21, z = 1.79, p = 0.074), which were monitored for up to 22 days after fungal exposure (Figure 4 insert).

Although fewer mosquitoes in the day 8 fungal exposure treatments survived to the end of the EIP, sporozoite prevalence (the proportion of mosquitoes with sporozoites in their salivary glands) was consistent across all treatment groups (Table 2). There was no evidence that sporozoite prevalence in the surviving mosquitoes was affected by either fungal exposure (F = 0.25, df = 1, p = 0.614) or exposure day (F = 0.37, df = 1, p = 0.544).

**Table 2:** **Summary of** ***Plasmodium falciparum*** **sporozoite prevalence**

**Exposure day**	**Fungal treatment**	**Proportion infectious (N)**
Day 8	Fungus	0.28 (39)
	Control	0.32 (84)
Day 11	Fungus	0.33 (43)
	Control	0.38 (34)

The proportion infectious *An. stephensi* mosquitoes is equal to the number of mosquitoes with successful sporozoite invasion of the salivary glands divided by the total number of mosquitoes dissected (N).

## Discussion

This study found no significant evidence that *B. bassiana* exposure affected malarial parasite development. With *P. yoelii*, there was no evidence that fungal co- or superinfection affected oocyst prevalence, oocyst intensity or the number of sporozoites per oocyst. Similarly, with *P. falciparum*, there was no evidence of an effect of fungal superinfection on sporozoite prevalence in surviving mosquitoes. Furthermore, there was no evidence in either experiment that malaria infection affected overall fungal virulence as measured by mosquito survival time.

Due to experimental constraints, many of the analyses associated with malaria infection parameters were based on small sample sizes. However, assuming α = 0.05 and β = 0.2, all of the analyses would have been expected to detect proportional differences of ≥0.8 except for the day 3 oocyst intensity assay (see Additional file 1). Although many of the analyses would have been insensitive to smaller impacts, low-level variation in malaria infection intensity or prevalence would likely be of limited importance in the context of transmission control. Mosquitoes with very few oocysts still become infectious, and the majority of mosquitoes that encountered fungus early in the malarial extrinsic incubation period (EIP) would not be expected to survive long enough to infect new hosts, particularly if higher fungal application rates were used [7]. For mosquitoes exposed late in the EIP, small reductions in sporozoite prevalence would likely have little impact on disease burden, particularly in high transmission areas where hosts may receive hundreds of infectious bites per year [62-64].

The results of this study generally support those of Blanford et al. [5], who performed an experiment similar to the day 0 *P. yoelii* experiment using a different species of malaria (*P. chabaudi*) but the same mosquito species and fungal strain. At the oocyst stage, neither study found evidence of an impact of fungal co-infection on oocyst prevalence or density. At the sporozoite stage, Blanford et al. [5] found that fungal co-infection significantly decreased the proportion of the initial (day 0) population which was both alive and infectious 14 days later. The sporozoite experiments in the current study differed from those in Blanford et al. [5] in two ways: sporozoite prevalence was evaluated in mosquitoes exposed to fungus late in the EIP (day 8 or day 11) and the lethal and non-lethal effects of fungal exposure were analysed separately. These analyses showed that, while fungal infection did significantly reduce mosquito survival in the day 8 exposures, there was no evidence of additional variation in sporozoite prevalence in the surviving population (i.e., those mosquitoes which were alive at the end of the EIP) for either exposure day. Given that there was also no evidence that malaria interacted with fungus to affect mosquito survival, these results suggest that the reductions in infectious mosquitoes in the late-exposure experiments were primarily mediated by mortality due to fungal infection rather than by interactions between *P. falciparum* and fungus.

Blanford et al. [5] did note an upward trend in daily mortality rate in mosquitoes co-infected with fungus and malaria around the sporozoite release stage which was absent in the treatment groups infected with fungus alone. The current study also found an apparent increase in daily mortality rate in the *P. yoelii* experiment starting at approximately day 12 in the ‘day 0 co-infection’ treatment relative to the ‘day 0 fungus alone’ treatment (see Additional file 2). However, the pattern was reversed in the equivalent super-infection treatments, with the ‘day 3 fungus alone’ treatment showing an increase in late stage daily mortality whereas the ‘day 3 superinfection’ treatment did not. Unfortunately, the very small sample sizes at the late stage of fungal infection make it difficult to interpret these patterns with great confidence, since differences of just one or two survivors would have led to large differences in proportional mortality.

The current study also agrees with a study by Fang *et al.* [65] using a different mosquito-fungus combination (*Anopheles gambiae* and *M. anisopliae*). That group reported no effect of late superinfection on ultimate prevalence or density of *P. falciparum* sporozoites in the salivary glands. These consistent results across different species and application methods suggest that the lack of interaction between fungal and malarial infections may be a general phenomenon among common fungal entomopathogens, though these dynamics can be altered via genetic modification [65].

Although malaria infection did not affect overall fungal virulence, mosquitoes exposed to fungus on the same day as the blood meal died marginally more quickly than those exposed three days later. Blood feeding greatly alters female physiology [66], and previous work has shown that there could be trade-offs between immune response and reproduction in mosquitoes [67]. Similar trade-offs could explain why mosquitoes might be more susceptible to fungal infection when they are actively digesting a blood meal. However, there is also evidence that blood feeding can temporarily increase resistance to fungal infection in *An. gambiae* and *Aedes aegypti* relative to individuals fed exclusively on glucose [68,69], though no such increase was observed in *An. stephensi* [7].

There are a number of additional factors that could influence mosquito-malaria-fungus interactions. For example, hydric and nutritional stress can increase mosquito mortality associated with malaria infection [51,53,70,71], and restricted diets are associated with a decrease in the mosquito melanization response [72]. The mosquitoes in this experiment were well-fed with easy access to sugar sources, potentially obscuring any resource competition or energetic tradeoffs involved in mounting an immune response that might occur under less favourable nutritional conditions. There is also natural variation between and among mosquito species to malaria infection [73,74], and the potential for complex effects of environmental variables, such as temperature, on mosquito immune function [75-77]. It is possible, therefore, that more diverse mosquito-parasite-pathogen interactions could be revealed under different contexts.

## Conclusion

Overall there was little evidence for impacts of the fungal pathogen, *B. bassiana*, on infections of either rodent or human malaria within the mosquito host or for any reciprocal effects of malaria infection on fungal virulence. These results suggest that, compared to factors such as biopesticide coverage, dose and substrate [7,10], malaria-fungus interactions will have a relatively small impact on the potential of this fungus to reduce malaria transmission.

## Additional files

Additional file 1:
**Power analyses for malaria infection parameters.**
Additional file 2:
**Summary of mean (± standard error) daily per cent mortality rate.**
