# Supplementary material for: Interactions between a fungal entomopathogen and malaria parasites within a mosquito vector
Source: Malar J. 2015 Jan 28;14:22. doi: 10.1186/s12936-014-0526-x (PMC4318179; doi:10.1186/s12936-014-0526-x)
Supplement: Additional file 1: — Power analyses for malaria infection parameters. [file 12936_2014_526_MOESM1_ESM.pdf]

Additional file 1 Power analyses for malaria infection parameters

A series of power analyses were performed to estimate statistical ability to detect differences in the various malarial infection parameters given the experimental sample sizes. For simplicity, comparisons were separated by exposure day. The analytical framework used depended on the parameter being evaluated.

The detectable effect sizes associated with the binomial analyses (*Plasmodium yoelii* oocyst prevalence and *Plasmodium falciparum* sporozoite prevalence) were estimated using the *pwr* package in R [1]. Assuming $\alpha = 0.05$, $\beta = 0.2$, and sample sizes equivalent to those used in each experiment, Cohen's effect size (h) [3] was calculated. Cohen's effect size is defined as:

$$h = 2 * \arcsin\left(\sqrt{P_0}\right) - 2 * \arcsin\left(\sqrt{P_0 - DP_0}\right)$$

where P_0 is the prevalence in one population and D is the proportional change in prevalence in the second population. Thus the detectable proportional change in prevalence could be calculated as follows:

$$D = 1 - \frac{\left[\sin\left(\arcsin\sqrt{P_0} - \frac{h}{2}\right)\right]^2}{P_0}$$

The analyses assumed that P_0 was equal to the prevalence in the control population. Results are summarized in Table A1.1.

The count data for the *P. yoelii* oocyst intensity and number of sporozoites per oocyst were both overdispersed, and traditional power analyses tend to overestimate power associated with small samples of overdispersed data [2]. For this reason, a Bayesian framework was employed in these

analyses. The *rnegbin* function in R [1] was first used to simulate a negative binomial distribution with a sample size, mean, and variance equivalent to the control population at one of the exposure time points (day 0 or day 3). The same procedure was then used to generate a second population equal in size to the corresponding fungal treatment group with its mean increased or decreased by a factor of 0.5-0.9. As in the experimental analysis, the two populations were compared using a negative binomial generalized linear model (GLM) with a log link. The procedure was repeated 5000 times, and power was estimated as the proportion of replicates in which the GLM detected a significant difference between the populations ($p < 0.5$, Table A1.2).

References

1. R Development Core Team: R: A language and environment for statistical computing. 2013.

2. Seavy NE, Quader S, Alexander JD, Ralph CJ: *Generalized Linear Models and Point Count Data: Statistical Considerations for the Design and Analysis of Monitoring Studies*. 2005:744–753.

3. Cohen J: *Statistical Power Analysis for the Behavioral Sciences*. 2nd edition. Lawrence Erlbaum Associates; 1988:590.

Table A1.1 Detectable effect size estimates for *Plasmodium yoelii* oocyst prevalence and *Plasmodium falciparum* sporozoite prevalence analyses

For each prevalence assay and exposure day, sample sizes for the control and treatment groups (‘N_0,’ ‘N_1’), control prevalence (‘P_0’), detectable effect size (‘h’), and corresponding proportional difference in prevalence between the control and treatment populations (‘D’) were reported. All analyses assumed $\alpha = 0.05$ and $\beta = 0.2$.

Parameter	Exposure day	N_0	N_1	P_0	h	D
P. yoelii oocyst prevalence	Day 0	20	20	0.90	0.89	0.42
	Day 3	20	20	0.70	0.89	0.62
P. falciparum sporozoite prevalence	Day 8	84	39	0.32	0.54	0.67
	Day 11	34	43	0.38	0.64	0.70

Table A1.2 Power estimates for analyses of *Plasmodium yoelii* oocyst intensity and number of sporozoites per oocyst

Exposure day determined the baseline sample sizes, mean, and variance in each set of analyses. Power was estimated for a variety of different proportional increases or decreases in mean ('D') for the analyses of oocyst intensity and number of sporozoites per oocyst (SPO) assuming $\alpha = 0.05$.

Exposure day	D	Oocyst intensity power		SPO power	
		Decrease	Increase	Decrease	Increase
Day 0	0.5	0.49	0.50	0.44	0.51
	0.6	0.60	0.64	0.55	0.63
	0.7	0.72	0.76	0.70	0.75
	0.8	0.81	0.85	0.88	0.84
	0.9	0.90	0.92	0.96	0.91
Day 3	0.5	0.29	0.18	0.45	0.49
	0.6	0.42	0.24	0.56	0.63
	0.7	0.56	0.28	0.71	0.75
	0.8	0.71	0.34	0.89	0.83
	0.9	0.88	0.40	0.97	0.90
