# Supplementary material for: Interactions between a fungal entomopathogen and malaria parasites within a mosquito vector
Source: Malar J. 2015 Jan 28;14:22. doi: 10.1186/s12936-014-0526-x (PMC4318179; doi:10.1186/s12936-014-0526-x)
Supplement: Additional file 2: — Summary of mean (± standard error) daily per cent mortality rate. [file 12936_2014_526_MOESM2_ESM.pdf]

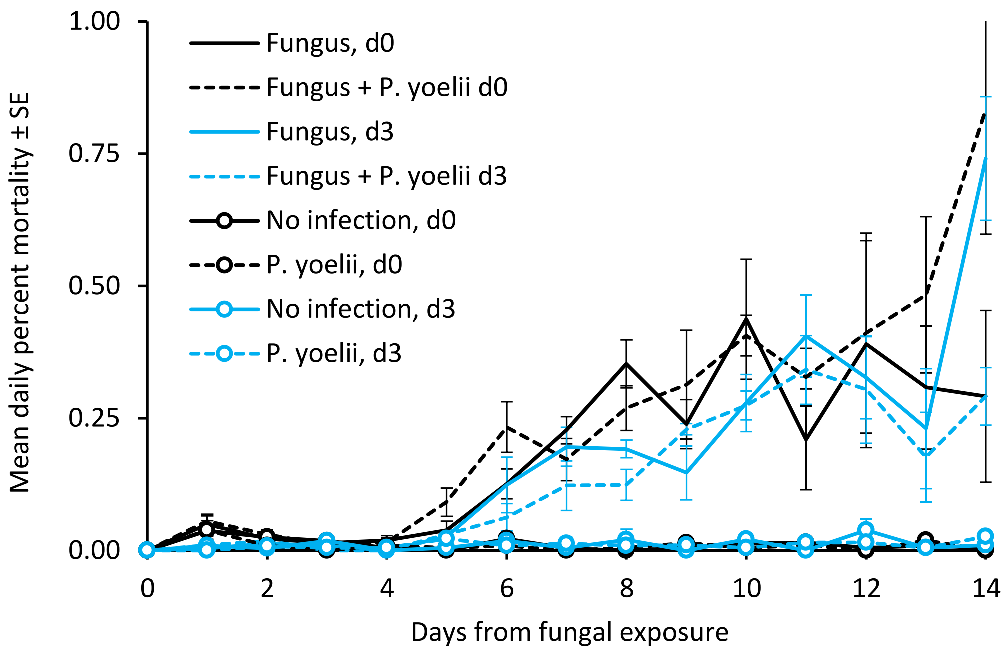

Additional file 2 Summary of mean (± standard error) daily percent mortality rate (e.g. number dead on a particular day divided by the number alive at the start of that day). *Anopheles stephensi* mosquitoes were fed either on *P. yoelii*-infected or control mice (indicated by line type), then exposed to tiles sprayed with *B. bassiana* conidial suspensions or blank oil (indicated by marker) either the same day as the blood meal (d0) or three days later (d3, indicated by line color).
